# Mastication and electrical activation in the masseter and anterior temporalis muscles of children and adolescents with osteogenesis imperfecta

**DOI:** 10.1590/2317-1782/e20240052en

**Published:** 2025-01-27

**Authors:** Andressa Colares da Costa Otavio, Mariana Vicereki Trescastro, Hilton Justino da Silva, Erissandra Gomes, Têmis Maria Félix

**Affiliations:** 1 Faculdade de Odontologia, Universidade Federal do Rio Grande do Sul - UFRGS - Porto Alegre (RS), Brasil.; 2 Universidade Federal de Pernambuco - UFPE - Recife (PE), Brasil.; 3 Serviço de Genética Médica, Hospital de Clínicas de Porto Alegre - HCPA - Porto Alegre (RS), Brasil.

**Keywords:** Speech, Language and Hearing Sciences, Stomatognathic System, Electromyography, Mastication, Osteogenesis Imperfecta, Child, Adolescent

## Abstract

**Purpose:**

to characterize mastication and electrical activation of the masseter and anterior temporalis muscles in children and adolescents with osteogenesis imperfecta (OI), and relate results to guided occlusion and occlusal interference.

**Methods:**

This observational, analytical cross-sectional study included 22 subjects divided into mild OI (MOI) (type 1) (n=15) and moderate-to-severe OI (MSOI) (types 3, 4, and 5) (n=7) groups. The Orofacial Myofunctional Evaluation with Scores (OMES) form was used to evaluate the clinical aspects of mastication. Surface electromyography was performed on the masseter and anterior temporalis muscles at rest for 10 seconds and during maximum intercuspation, spontaneous chewing, and instructed chewing on the right and left sides. Additionally, the activation index and muscle symmetry were measured.

**Results:**

a preferentially unilateral chewing pattern was observed in 12 (54.5%) participants. Masticatory patterns did not influence electrical activation during any of the tasks, nor did occlusal guidance during maximum intercuspation or mastication. The percentage of muscle activation during maximal intercuspation approached half of the total activation during spontaneous chewing. In muscle activation indices, the MSOI group presented more atypical scores, while the MOI group scores seemed to be in line with reference values. The symmetry indices seemed to correspond to reference values, but the standard deviation and minimum and maximum values pointed to asymmetric results.

**Conclusion:**

This study found that the OI population presented muscle imbalances, but the results did not allow us to define one pattern of change.

## INTRODUCTION

Osteogenesis Imperfecta (OI) is a rare genetic condition, with a prevalence in South America of 0.74 cases per 10,000 live births^(1)^. It is a connective tissue disorder with wide molecular variability but with similar phenotypes^(2)^. The phenotypes range from mild to lethal forms^(3)^. Clinically, they are classified into five types: type 1 are mild and non-deforming phenotypes, type 2 are perinatal lethal, type 3 are severe phenotypes and type 4 are moderate phenotypes^(4,5)^. The best-known signs and symptoms of OI include bone fragility, skeletal deformities, easy fractures, short stature, hearing loss, blue sclera, and loose ligaments^(4,6-8)^.

Oral changes, including dentinogenesis imperfecta, hypo and oligodontia, taurodontism, second molar retention, and occlusal problems have also been described^(9-11)^. Changes in the stomatognathic system have been described in few studies. In children with congenital bone fragility, there were acoustic symptoms from the temporomandibular joints more often than in healthy children^(12)^. Problems with swallowing, sucking and temporomandibular joint function were also observed^(13)^. No studies with quantitative assessments of the stomatognathic system were found, leaving a gap for future research.

Orofacial myofunctional assessments can be expanded to include measurable instrumental diagnostic methods to quantitatively evaluate the functioning of orofacial muscles^(14,15)^. Surface electromyography (sEMG) is a valuable technique for studying movement and the mechanisms involved in neuromuscular physiology, and for diagnosing neuromuscular disorders. However, there are some limitations. It can only be used to assess superficial muscles and, in some cases, it is impossible to isolate and activate single muscles. Even so, sEMG has the advantage of being a non-invasive, easy technique^(16)^. To date, no reports have been found describing the use of surface electromyography to evaluate the stomatognathic system in OI population samples.

Thus, this study aimed to characterize mastication and electrical activation of the masseter and anterior temporalis muscles in children and adolescents with osteogenesis imperfecta and relate results to guided occlusion and occlusal interference, as well as to present findings regarding activation indices and muscle symmetry.

## METHODS

This cross-sectional study was approved by the research ethics committees of the Universidade Federal do Rio Grande do Sul (UFRGS) and the Hospital de Clínicas de Porto Alegre (HCPA), approval number 3.526.427. Research participants and their guardians agreed to participate in this study and signed informed consent forms.

### Participants

Participants were recruited at the renowned special reference center for Osteogenesis Imperfecta at HCPA, in southern Brazil. They were included in the study if they had a clinical diagnosis of OI and were between 6 and 19 years of age. Subjects were excluded if they has undergone speech therapy over the previous six months, or had a medical history of surgery, tumors, or head or neck trauma.

Convenience sampling was based on the inclusion criteria. The sample size estimate, based on the number of registered patients, showed that seventy patients were eligible to participate. Among these patients, 67.14% (47) were type 1 OI while 32.85% (23) were types 3, 4, and 5. Subjects were grouped according to severity: the mild OI (MOI) group included participants with a clinical classification of type 1 OI, whereas the moderate-to-severe OI (MSOI) group included participants with types 3, 4, and 5 clinical classifications^(11)^.

To calculate the sample size, the estimated prevalence for both groups (MOI vs. MSOI), a significance level of 5% (α), and a sampling error (d) of 0.16 was adopted. Because it is a rare disease, a finite population correction factor was also used. There were 22 cases in the final minimum sample size: 15 MOI and 7 MSOI.

The study protocol consisted of the following steps: a) after a routine consultation at the outpatient clinic, individuals were invited to participate in the study; b) those who accepted were referred to perform the study evaluations; c) clinical evaluation was performed with OMES protocol the items guided occlusion and occlusal interferences during lateral excursions were analyzed^(17,18)^; d) an orofacial myofunctional evaluation of chewing simultaneously with surface electromyography during chewing tasks to meet the study objectives. The chewing was performed with video camera recording for later analysis. (Supplementary Material chart 2).

### Orofacial myofunctional assessment

The OMES orofacial myofunctional assessment scoring form^(17)^ was used. This tool is composed of items that verify different aspects regarding the appearance and posture of structures, the mobility of orofacial structures, and orofacial functions. For this study, only the item related to mastication was used.

For the clinical evaluation, spontaneous chewing, guided occlusion, and occlusal interferences during lateral excursions were analyzed^(17,18)^. During the entire assessment, each participant remained seated with their feet flat on the floor, about 1m away from a video camera mounted on a tripod. The same experienced evaluator – who had had previous training in using the OMES form - tested all participants. A Bono® sandwich cookie was offered to each participant with the following spoken instruction: “Eat this cookie as you usually do”. There was no interference from the evaluator while the patient ate.

### Surface electromyography

This part of the assessment was recorded with Miotec® equipment, Miotool software, four input channels. A 20Hz high pass filter and a 500Hz low pass filter were similarly used. Pairs of Kendall™ pediatric disc-shaped electrodes were spaced 20 mm apart and were adhered to the bellies of the right and left masseter muscles as well as the right and left anterior bellies of the temporalis muscle. Participants with beards were asked to remove them. Surface ElectroMyoGraphy for the Non-Invasive Assessment of Muscles (SENIAN) recommendations were followed^(19)^.

The electromyographic signals from the masseter and anterior temporalis muscles were normalized using a maximum voluntary contraction (MVC), which is considered 100% electrical muscle activation. The Pernambuco et al.^(20)^ method was used to capture the MVC. The patients were previously trained. The verbal command to begin this test was “Clench your teeth together as hard as you can”, and participants were encouraged with further verbal stimuli such as “Squeeze, squeeze, squeeze”.

After collecting the MVC, the following tasks were performed for assessment:

- Rest for 10s: each participant was instructed to remain completely still for 10s. Additional guidance was given so that they did not move their tongues or swallow saliva;
- maximum intercuspation: each patient clenched their teeth three times for five seconds as firmly as possible, no cotton rolls. There was a rest interval of 10 seconds between each contraction. The patients were previously trained. The verbal command to begin this test was “Clench your teeth together as hard as you can”, and participants were encouraged with further verbal stimuli such as “Squeeze, squeeze, squeeze”;
- spontaneous chewing with a Bono® sandwich cookie – this particular food stimulus was offered during the test because it was validated by the OMES form we used to evaluate the masticatory function^(17)^. Participants were given the following verbal command: “Eat this cookie as you do at home”;
- instructed chewing on the right side with a Bono® sandwich cookie - after a two-minute rest, each participant was instructed to chew on only the right side of their mouth. They were given another cookie and the following verbal command: “Chew on the right side only”. During this task, the evaluator repeated a few times “only on the right side” while pointing to the patient's right;
- instructed chewing on the left side with a Bono® sandwich cookie - after a two-minute rest, each participant was instructed to chew on only the left side of their mouth. They were given another cookie and the following verbal command: “Chew on the left side only”. During this task, the evaluator repeated a few times “only on the left side” while pointing to the patient's left.

To analyze and read electromyographic signals from the 5-second maximum intercuspation task, the first and last seconds were excluded and the mean (%) of the 3-second electrical activation period was recorded. After that, the arithmetic mean of the three repetitions was calculated and this was the value used for analysis. For the chewing tasks, the interval analyzed was between the first incision and the last swallow^(21)^. The mean (%) of the electrical activation recorded with the normalized electromyographic signal was documented^(21)^.

The muscle activation index and the muscle asymmetry index were also analyzed. The muscle activation index measures the relative contribution between the muscles and makes it possible to detect the pair of prevalent muscles^(22)^. The positive results of this equation indicate a predominance of masseter muscle activation and negative values for the anterior temporalis muscles^(22,23)^. This formula was proposed in a previous study^(23)^. The muscle asymmetry index make it possible to detect the symmetry between the muscles on the right and left sides. Positive values indicate a predominance of right-side muscle activation and negative values for the left-side muscle^(22,23)^.

All tests and interpretations were performed in the same environment, by the same evaluator who had been previously trained in clinical and research practice. The chewing tasks and sEMG were recorded with a video camera simultaneously to aid with the subsequent chewing analyses. All tests and interpretations were performed in the same environment, by the same evaluator who had been previously trained in clinical and research practice. The chewing tasks and sEMG were recorded with a video camera simultaneously to aid with the subsequent chewing analyses.

### Statistical analysis

The data were statistically treated with the help of the SPSS® statistical program, version 20.0 for Windows®. A significance level of 5% was used for all tests. That is, the null hypothesis was rejected when the p-value was less than or equal to 0.05. Results were presented in absolute and relative distributions (n - %), as well as measures of central tendency (mean and median) and variability (standard deviation and amplitude), with a Shapiro-Wilk symmetry test. The Mann-Whitney U test and Student's t-test were used to compare continuous variables between two independent groups.

## RESULTS

There were 22 participants, 15 in the MOI group and 7 in the MSOI group. The mean age of the total sample was 12.09±4.3 years, 12.87±3.6 years in the MOI group, and 10.43±5.5 years in the MSOI group. In the total sample, 12 (54.6%) were female and 10 (45.4%) were male. In the MOI group, 6 (40%) were female and 9 (60%) were male while in the MSOI group, 6 (85.7%) were female and 1 (14.3%) male.

No age groups were established, nor division by sex, therefore statistical analysis was applied to verify whether the results were influenced by age and sex. Spearman's correlation was used to compare age on the electromyographic evaluation for the masseter muscle (r = -0.104; p = 0.646) and for the anterior temporal muscle (r = 0.183; p = 0.414) and did not show statistically significant results. Therefore, there is no evidence in this sample that age can influence or impact the muscular electrical activity in the masseter and anterior temporal muscles. The electrical activity for these muscles was also compared according to sex. According to the Student's t-test, in relation to sex, no statistically significant results were detected. According to the information collected, it was found that, both for the masseter muscle (Female: 18.16±4.25 vs. Male: 20.70±5.73; p=0.248), and for the anterior temporal muscle (Female: 18.46±5.29 vs. Male: 22.40±6.03; p=0.118), the male sex presented higher averages, however the differences observed were not representative. The model was calculated using the values of electrical activation (%) registered during the maximum intercuspation task, and the arithmetic mean was calculated using the results of the right and left sides.

In the MOI group, four (26.7%) participants presented a rotary chewing pattern, nine (60%) a preferentially unilateral pattern, and two (13.3%) a chronic unilateral pattern. In the MSOI group, three (42.9%) presented a rotary chewing pattern, three (42.9%) a preferentially unilateral pattern, and one (14.3%) a simultaneous bilateral pattern. In the total sample, guided occlusion was observed in 12 (55.5%) participants, both on the right and on the left sides. There was no occlusal interference with opposing teeth for 13 (59.1%) on their right side and 14 (63.6%) on their left side. In the MOI group, 9 (60%) presented guided occlusion both on the right and the left sides. Ten subjects (66.7%) exhibited no occlusal interference with opposing teeth on the right and 12 (80%) on the left. In the MSOI group, 3 (42.9%) showed guided occlusion both on the right and on the left; 3 (42.9%) demonstrated no occlusal interference with opposing teeth on the right, and 2 (28.6%) on the left. The Kappa coefficient of agreement was used and showed an intra-rater agreement classified as almost perfect [Kappa = 0.803; Standard error = 0.015; p<0.001].

Table 1 presents and compares the results for the total sample, and between the OI groups, with regard to the measures of central tendency and variability of the electrical activation at rest (10s), during maximum intercuspation, spontaneous chewing, and instructed chewing (right and left), for the masseter and anterior temporalis muscles on the right and left side. According to the results, a statistically significant difference was detected for the rest task for the right and left masseter muscles. The mean in the MSOI group was higher than the estimate in the MOI group. On the maximal intercuspation and left-sided chewing tasks, we found a significant difference between groups with regard to the right anterior temporalis muscle. The MOI group demonstrated a higher average than the MSOI group. As for the spontaneous chewing task, the right masseter muscles showed a significantly higher mean in the MSOI group than in the MOI group. With other variables, the differences between the means were not representative.

**Table 1:** Measures of central tendency and variability of the right and left masseter and anterior temporalis muscles at rest (10s), and during maximum intercuspation, spontaneous chewing, and instructed chewing (right and left) for the total sample and each OI group

**Variables** ^A^	**Sample total (n=22)**					**OI Groups**										
						**MOI (n=15)**					**MSOI (n=7)**					
	**Mean**	**SD**		**Quartile**		**Mean**	**SD**		**Quartile**		**Mean**	**SD**		**Quartile**		**p** ^¥^
			**1º**	**2º**	**3º**			**1º**	**2º**	**3º**			**1º**	**2º**	**3º**	
**Surface electromyography at rest (% MVC in µV)**																
Right masseter	1.07	0.68	0.49	0.94	1.60	0.85	0.54	0.39	0.82	1.10	1.55	0.74	0.83	1.67	2.32	0.038
Left masseter	1.11	0.82	0.50	0.81	1.42	0.85	0.66	0.48	0.74	0.88	1.65	0.92	0.81	1.28	2.77	0.012
Right anterior temporalis muscle	1.80	0.90	1.06	1.47	2.46	1.81	0.86	1.04	1.48	2.55	1.79	1.05	1.08	1.46	2.43	0.916
Left anterior temporalis muscle	2.27	1.50	0.95	2.22	3.05	2.07	1.37	0.93	2.05	2.88	2.68	1.79	1.53	2.43	3.66	0.503
**Surface electromyography maximum intercuspation (% MVC in µV)**																
Right masseter	19.96	5.49	16.35	19.85	22.30	19.14	3.29	16.40	19.41	21.64	21.73	8.65	14.50	21.55	30.68	0.549
Left masseter	18.80	5.57	14.46	18.35	23.48	18.29	4.50	14.47	17.34	23.31	19.89	7.71	14.20	19.07	24.23	0.751
Right anterior temporalis muscle	19.15	6.94	13.34	18.46	22.68	21.27	7.05	14.33	20.64	24.33	14.61	4.18	12.07	14.15	18.43	0.026
Left anterior temporalis muscle	21.92	5.87	18.17	21.67	25.16	22.78	5.39	18.42	22.55	25.39	20.07	6.85	14.01	20.37	23.57	0.341
**Surface electromyography spontaneous chewing (% MVC in µV)**																
Right masseter	8.84	3.21	6.39	7.94	12.05	7.94	3.16	6.14	6.75	8.06	10.78	2.54	8.40	11.65	12.70	0.032
Left masseter	8.92	3.53	6.32	8.78	12.03	8.32	3.18	6.08	7.18	11.93	10.19	4.15	6.58	10.70	13.60	0.332
Right anterior temporalis muscle	9.07	3.19	6.72	8.92	10.93	9.78	3.17	7.18	9.35	11.45	7.56	2.89	4.62	7.34	10.37	0.129
Left anterior temporalis muscle	10.44	3.06	7.14	11.07	13.13	10.49	3.26	7.15	10.99	13.44	10.33	2.82	7.09	11.15	12.39	0.891
**Surface electromyography instructed chewing right (% MVC in µV)**																
Right masseter	10.76	4.32	7.36	9.87	13.28	9.85	4.25	6.72	9.64	11.48	12.73	4.07	7.88	13.22	15.66	0.091
Left masseter	7.35	3.24	4.41	6.91	9.87	6.54	2.57	4.15	6.28	8.46	9.08	4.02	5.02	9.46	13.36	0.142
Right anterior temporalis muscle	9.09	2.74	7.02	8.85	11.15	9.54	2.37	7.91	8.93	11.08	8.12	3.39	5.17	7.03	11.34	0.298
Left anterior temporalis muscle	10.30	3.22	7.74	10.55	12.80	10.65	3.41	8.90	11.23	12.93	9.55	2.84	7.24	9.31	11.99	0.490
**Surface electromyography instructed chewing left (% MVC in µV)**																
Right masseter	22	7.79	3.61	5.09	6.64	15	7.27	3.91	5.12	6.05	7	8.89	2.79	4.99	9.70	0.368
Left masseter	10.96	4.12	9.11	9.82	15.02	10.35	4.05	8.51	9.62	14.44	12.27	4.27	9.31	13.78	15.18	0.407
Right anterior temporalis muscle	8.60	3.19	5.67	8.92	11.21	9.59	2.98	7.17	10.37	11.99	6.48	2.66	4.63	4.96	9.25	0.039
Left anterior temporalis muscle	11.29	3.66	8.14	11.40	13.74	11.12	3.43	8.18	11.34	14.12	11.64	4.37	6.94	12.35	13.53	0.945

¥ Mann-Whitney U test comparing the MOI and MSOI groups;

A asymmetrical distribution of variables (Shapiro Wilk; p<0.05)

**Caption:** MVC = maximum voluntary contraction; µV = micro volts; SD = standard deviation; MOI = mild osteogenesis imperfecta group; MSOI = moderate-to-severe osteogenesis imperfecta group

Comparisons between the variables in Table 1 with the masticatory patterns could not confirm a relationship between the electrical activation generated during the different tasks and the masticatory patterns (Appendix A – 1). For the statistical analysis, chewing types were grouped in a binary manner: rotary chewing versus other types of chewing. Regarding the guided occlusion, there were significant results for the resting task. This shows that, when there is no canine-guided occlusion, there is greater electrical activation in the masseter and anterior temporalis muscles at rest (Appendix B – 2). The comparison with occlusal interference showed that when this phenomenon occurred on the working side, there was increased electrical activation at rest and while chewing (Appendix C – 3).

Table 2 presents the muscle activation rates for the total sample and each OI group. During the rest task, both groups showed a predominance of anterior temporalis muscle activation. However, the MOI group showed more use of these muscles than the MSOI group. On the other tasks, the MOI group presented a muscle activation index with a predominance of anterior temporalis muscle involvement, while the MSOI group presented an index with a predominance of masseter muscle involvement. All data were statistically significant (p<0.05).

**Table 2:** Muscle activation index of the masseter and anterior temporalis muscles at rest (10s), and during maximum intercuspation, spontaneous chewing, instructed chewing on the right side, and instructed chewing on the left side, for the total sample and each OI group

**Tasks**	**Muscle Activation Index (%)**																					
	**Sample total (n=22)**							**OI Groups**														
								**MOI (n=15)**							**MSOI (n=7)**							
	**X**	**SD**	**Min**	**Max**	**Quartile**			**X**	**SD**	**Min**	**Max**	**Quartile**			**X**	**SD**	**Min**	**Max**	**Quartile**			**p**
					**1º**	**2º**	**3º**					**1º**	**2º**	**3º**					**1º**	**2º**	**3º**	
**At rest**	-30.23	24.54	-81.00	9.00	-50.00	-25.00	-11.25	-37.27	23.45	-81.00	-8.00	-50.00	-40.00	-15.00	-15.14	20.86	-51.00	9.00	-28.00	-14.00	7.00	0.029
**Maximum Intercuspation**	-2.36	13.83	-30.00	29.00	-12.50	0	5.25	-17.13	12.18	-30.00	13.00	-15.00	-6.00	2,.00	7.86	12.03	-8.00	29.00	2.00	4.00	18.00	0.022
**Spontaneous chewing**	-5.59	15.17	-35.00	24.00	-17.00	-6.50	7.25	-11.47	13.11	-35.00	15.00	-22.00	-12.00	-4.00	7.00	11.52	-14.00	24.00	2.00	8.00	13.00	<0,001
**Instructed chewing**																						
**Right**	-4.95	15.82	-36.00	24.00	-18.50	-4.50	7.00	-11.73	13.35	-36.00	21.00	-20.00	-14.00	-4.00	9.57	9.95	-4.00	24.00	2.00	7.00	21.00	<0.001
**Left**	-4.55	15.22	-38.00	27.00	-16.25	-4.00	6.00	-9.87	14.26	-38.00	21.00	-18.00	-7.00	-1.00	6.86	10.65	-7.00	27.00	-1.00	6.00	11.00	0,003

**Caption:** Mann Whitney U test MOI x MSOI groups; MOI = mild osteogenesis imperfecta group; MSOI = moderate-to-severe osteogenesis imperfecta group; X = mean; SD = standard deviation; Min = minimum; Max = maximum

Table 3 presents the muscle asymmetry indices for the total sample and each OI group. There was only a significant difference between the OI groups for anterior temporalis muscle activation during the left-sided chewing task. The MSOI group was more asymmetrical than the MOI group. Table 3 shows that, even with no significance between the groups, in general, the MSOI group exhibited higher mean asymmetry values than the MOI group.

**Table 3:** Muscle asymmetry index of the masseter and anterior temporalis muscles at rest (10s), and during maximum intercuspation, spontaneous chewing, chewing on the right side, and chewing on the left side for the total sample and each OI group

**Tasks**	**Muscle Asymmetry Index (%)** ^A^														
Groups	**T**				**p¥**	**MMA**				**p¥**	**ATMA**				**p** ^¥^
	**X**	**SD**	**Min**	**Max**		**X**	**SD**	**Min**	**Max**		**X**	**SD**	**Min**	**Mx**	
**At rest**															
Total	-4.05	20.12	-42.00	35.00		-0.41	17.92	-25.00	40.00		-4.95	26.39	-54.00	56.00	
MOI	-1.27	16.07	-31.00	35.00	0.341	0.20	18.91	-23.00	40.00	1.000	-0.47	19.65	-35.00	39.00	0.129
MSOI	-10.00	27.45	-42.00	35.00		-1.71	16.93	-25.00	29.00		-14.57	37.13	-54.00	56.00	
**Maximum intercuspation**															
Total	-2.00	9.08	-18.00	13.00		3.27	12.55	-34.00	21.00		-7.82	11.60	-34.00	15.00	
MOI	-0.80	7.90	-18.00	11.00	0.341	3.00	12.97	-34.00	19.00	0.944	-4.60	10.14	-24.00	15.00	0.078
MSOI	-4.57	11.47	-18.00	13.00		3.86	12.56	-14.00	21.00		-14.71	12.24	-34.00	0.00	
**Spontaneous chewing**															
Total	-3.14	10.49	-21.00	15.00		0.50	14.90	-35.00	26.00		-7.36	16.42	-52.00	16.00	
MOI	-2.33	10.93	-21.00	15.00	0.548	-1.87	16.20	-35.00	20.00	0.596	-3.07	14.05	-32.00	16.00	0.104
MSOI	-4.86	10.09	-21.00	12.00		5.57	10.98	-8.00	26.00		-16.57	18.39	-52.00	2.00	
**Instructed chewing**															
**Right**															
Total	6.45	12.89	-16.00	33.00		19.36	17.98	-14.00	47.00		-5.73	17.21	-29.00	26.00	
MOI	6.73	14.45	-16.00	33.00	0.860	19.60	19.61	-14.00	47.00	0.972	-3.80	18.53	-28.00	26.00	0.359
MSOI	5.86	9.65	-3.00	22.00		18.86	15.28	-1.00	36.00		-9.86	14.36	-29.00	9.00	
**Left**															
Total	-14.82	12.25	-44.00	-1.00		-17.64	17.01	-46.00	8.00		-13.64	19.86	-64.00	18.00	
MOI	-12.20	9.41	-32.00	-1.00	0.274	-19.20	17.37	-46.00	7.00	0.480	-7.47	15.98	-39.00	18.00	0.038
MSOI	-20.43	16.28	-44.00	-3.00		-14.29	17.00	-41.00	8.00		-26.86	22.16	-64.00	-3.00	

¥ Mann-Whitney U test comparing the MOI and MSOI groups;

A asymmetrical distribution of variables (Shapiro Wilk; p<0.05)

**Caption:** T = total asymmetry index; MMA = masseter muscle asymmetry index; ATMA = anterior temporalis muscle asymmetry; MOI = mild osteogenesis imperfecta group; MSOI = moderate-to-severe osteogenesis imperfecta group; X = mean; SD = standard deviation; Min = minimum; Max = maximum

**Figure 1:**
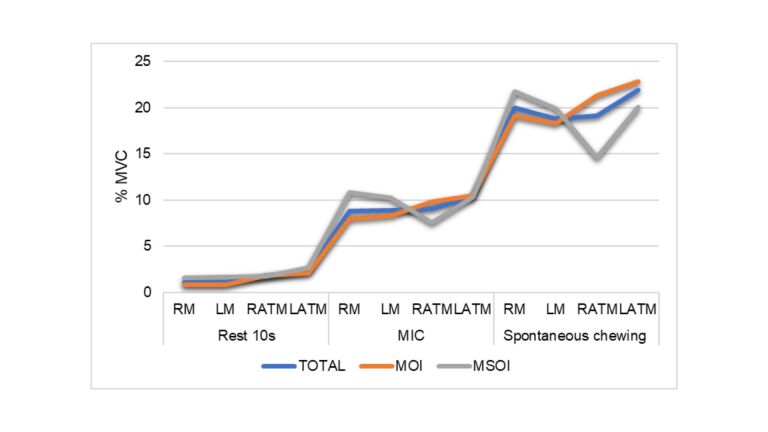
Percentage of maximum voluntary contact in the tasks of rest for 10s, maximum intercuspation and spontaneous chewing

**Figure 2:**
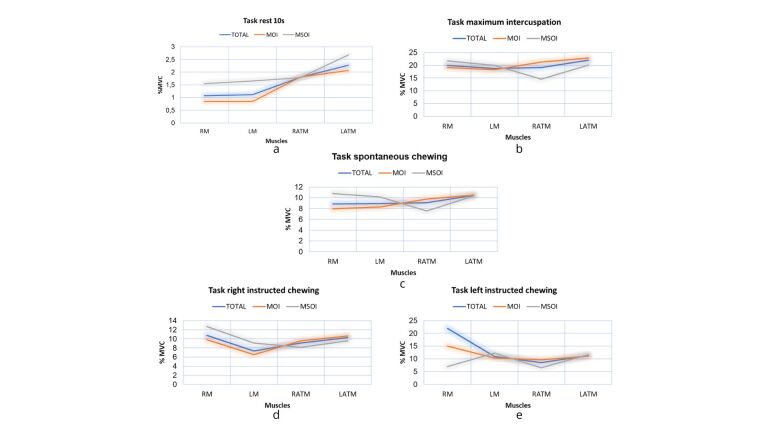
Percentage of maximum voluntary contraction for tasks at rest for 10s (a), maximum intercuspation (b), spontaneous chewing (c), right instructed chewing (d) and left instructed chewing (e)

Figure 1 illustrates the MVC percentage at rest, and during the maximum intercuspation and spontaneous chewing tasks, for the total sample and each OI group. The percentage of activation during maximum intercuspation neared half of the percentage during spontaneous chewing. Figure 2 illustrates the percentage of muscle activation during the different tasks per group, in detail. Figure 2a shows that, at rest, the MSOI group presented more muscle activation than the MOI group and the total sample. Figure 2b demonstrates that the MSOI group exhibited more masseter activation than the rest of the sample, and less activation in the anterior temporalis muscle. The same was true for spontaneous chewing (2c) and right-sided instructed chewing (2d). During left-sided instructed chewing, the masseter and right anterior temporalis muscles in the MSOI group exhibited lower levels of activation (2e).

MVC = maximum voluntary contraction; MIC = Maximum intercuspation; RM = right masseter muscle; LM = left masseter muscle; RATM = right anterior temporalis muscle; LATM = left anterior temporalis muscle; MOI = mild osteogenesis imperfecta group; MSOI = moderate-to-severe osteogenesis imperfecta group

MVC = maximum voluntary contraction; MIC = Maximum intercuspation; RM = right masseter muscle; LM = left masseter muscle; RATM = right anterior temporalis muscle; LATM = left anterior temporalis muscle; MOI = mild osteogenesis imperfecta group; MSOI = moderate-to-severe osteogenesis imperfecta group

## DISCUSSION

Among the repercussions that have been reported for the stomatognathic system in patients with OI, the acoustic symptoms of temporomandibular disorders (TMD) have been described more frequently in children with the disease^((12))^, in addition to changes in eating and swallowing^(13)^.

In this sample of children and adolescents with OI, when the right and left masseter muscles were assessed at rest, there was a statistical difference between the groups. The MSOI participants presented a higher activation average than the MOI participants. The detection of muscle activation during rest is justified because, even at complete rest, muscles do not lose their tone^(24)^. Thus, the efficient thermodynamics and the architecture for the motor control to perform tasks are safely maintained, for static or dynamic actions^(25)^. Regarding the tasks of maximum intercuspation, left-sided chewing, and spontaneous chewing, there were differences between the OI groups but there were no clinical findings of mastication (i.e. type, guided occlusion, and occlusal interferences) that could be correlated with these data. There was apparent disorganization of muscle synergies in this population.

A rotary chewing pattern could not be associated with electrical muscle activation during the different tasks we assessed. A study with chewing gum reported differences in muscle activation between distinct chewing pattern^(26)^. The rotary pattern is the normal, mature physiological one. However, for this pattern to develop, the mandibular muscles must function adequately and in harmony with other orofacial structures - such as the lips, tongue, and cheeks - as well as the temporomandibular joints, craniofacial morphology, occlusion, and general oral health. When these conditions are not met, adaptations or compensations arise^(27)^.

In this study, occlusal interference on the working side was related to electrical activation at rest and during chewing tasks. In a normal occlusal relationship during lateral movements, the canine teeth on the working side (i.e. the side of the mandibular excursion) come into contact and disocclude the opposing teeth (on the opposite side to the mandibular excursion)^(28)^. When occlusal interferences occur between teeth on the opposite side, they can negatively affect the chewing function^(18)^ and may be associated with craniomandibular disorders^(29)^. Non-physiological occlusion can change the proprioceptive and periodontal stimuli that are sent to the central nervous system. In an apparent attempt to avoid interference, these changes will modify activation sequences, and the duration and number of active motor units^(29)^.

The percentage of muscle activation during maximal intercuspation was lower than the percentage of the spontaneous chewing task. Maximum intercuspal movement is a static one that occurs only in the sagittal plane. During mastication, however, for dynamic movement to occur, each sagittal, vertical, and horizontal axis must tilt to accommodate the movement occurring on the other axes. This requires complex control of the neuromuscular system to prevent damage to any oral structures^(28)^. The wide distribution of type I collagen in the temporomandibular joint is well documented^(30,31)^. Since this protein is affected by OI, potential instability in the maxillomandibular complex can be expected. Furthermore, there is evidence, both in mouse models and in human studies, that some forms of OI have been associated with low muscle mass and function^(32)^.

Regarding the greater activation in the MSOI group at rest, another study that used the MVC% to analyze the influence of TMD on the electromyographic activation of the masseter and temporalis muscles of adolescents found that the amplitude of the EMG activation was significantly greater at rest in the moderate-to-severe TMD group than the mild or no TMD groups^(33)^.

The greater activation of the anterior temporalis muscles during spontaneous chewing and instructed chewing on the right side in the MOI group can be justified by the vertical elevation of the mandible caused by the contraction of the anterior portion of this muscle^(28)^, to compensate for any instability in the complex. The anterior portion of the temporalis muscle has a stabilizing function^(34)^. The greater activation of the temporalis muscles in relation to the masseter muscles is justified by their compensatory action^(26)^.

As for the muscle activation indices, at rest, both groups showed a predominance of anterior temporalis muscle use. However the MOI group showed even more use of this muscle when compared to the MSOI group. This data corroborates the literature that describes 66% of individuals as having a predominance of anterior temporalis muscle activation during rest (for 2 minutes). In addition, only 5% did not exhibit the predominance of any muscle^(23)^. During the other tasks, the MOI group presented a muscle activation index with predominant anterior temporalis muscle activation. This finding also corroborates the literature that describes the maximal voluntary contraction task and reports that the anterior temporalis muscle was more active in 80% of the individuals. The MSOI group, however, presented an index with predominant masseter muscle activation. By using the rest and MVC scores, we inferred that the MSOI group presented more signs of changed muscular activation.

The muscle asymmetry indices only showed a difference between the groups in anterior temporalis muscle activation while chewing. The MSOI group was more asymmetric. Even with no differences in other indices, the MSOI group presented higher mean asymmetry values than the MOI group. In electromyography, the neuromuscular balance underlying healthy orofacial structures is expressed as symmetrical muscle activation, and perfect symmetry is equal to 0%^((29))^. Nevertheless, most individuals present asymmetrical muscle activation^(23)^. A rest, only about 10% may present an asymmetry index equal to zero^(23)^. The reference values in a sample of adults reported higher asymmetry averages than those of the rest (2 minutes) and MVC tasks in our study sample^(23)^.

It drew attention to the fact that the standard deviation for muscle asymmetry indices was much higher for total asymmetry and anterior temporalis muscle asymmetry^(23)^. The minimum and maximum values of this sample are similarly noteworthy since they show very asymmetric results. When there is a prevalence of electromyographic activation, the load reaction on the condyles is greater on the opposite side. During a balanced performance, the ergonomics of the system are not required to make trade-offs. Disruptions in balance caused by changes that overload the system can result in abnormal loads on the periodontium and erosion, thus increasing component wear. In addition, the masticatory muscles exert a considerable influence on craniofacial morphology, affecting the growth of the bones into which they are inserted^(29)^.

This is the first known study to have performed such assessments in a population of children and adolescents with OI. Although the required sample size was achieved in a rare disease, it was not possible to divide into groups by facial pattern, molar relationship, occlusion types, and dentition types, and this was a limitation for this study. The data indicate that there is muscle imbalance among the OI population, but did not allow us to define a pattern of change. Performing multiple assessments of large samples in populations with rare diseases is a particularly difficult task. Nevertheless, future studies with a larger sample and a control group are recommended for further analyses. This includes investigations into subdivisions between the different occlusal classifications, facial typology, and molecularly identified OI types.

## CONCLUSIONS

In this sample, the most common chewing patterns were preferential unilateral and alternating bilateral. Regarding the spontaneous chewing task, the right masseter muscles presented a significantly higher mean in the MSOI group than in the MOI group.

Masticatory patterns could not be associated to electrical activation during tasks, and guided occlusion did not seem to influence electrical activation during maximum intercuspation and mastication. When there is no canine-guided occlusion, there is greater electrical activation in the masseter and anterior temporal muscles at rest. When there is occlusal interference on the working side, there was an increase in electrical activation at rest and during chewing. The percentage of muscle activation during maximum intercuspation was almost half of the percentage of activation during the spontaneous chewing task.

The muscle activation indices suggest that subjects with moderate-to-severe OI present more changes in muscle activation, while those with milder cases achieved scores similar to the reference values. Although the mean values of the symmetry indices were in accordance with the reference values, the standard deviation for total asymmetry and anterior temporalis muscle asymmetry were much higher, with the minimum and maximum scores indicating very asymmetric results.

## Appendix A

**Table S1:** Measures of central tendency and variability of the masseter and anterior temporalis muscles at rest (10s), and during maximum intercuspation, spontaneous chewing and chewing on the right and left sides, according to chewing pattern

**Variables** ^A^	**Chewing pattern**	**Descriptive measures**						
		**n**	**Mean**	**SD**	**Quartile**			**p** ^¥^
					**1º**	**2º**	**3º**	
**Surface electromyography at rest (% MVC in µV)**								
Right masseter	Rotary	7	1.10	0.67	0.54	0.83	1.70	0.778
	Other	15	1.06	0.71	0.39	0.99	1.57	
Left masseter	Rotary	7	1.00	0.48	0.74	0.78	1.28	0.860
	Other	15	1.16	0.95	0.48	0.81	1.72	
Right anterior temporalis muscle	Rotary	7	1.64	0.79	1.06	1.38	2.43	0.647
	Other	15	1.88	0.96	1.04	1.66	2.55	
Left anterior temporalis muscle	Rotary	7	1.85	0.86	0.96	1.98	2.55	0.549
	Other	15	2.46	1.71	0.93	2.39	3.59	
**Surface electromyography maximum intercuspation (% MVC in µV)**								
Right masseter	Rotary	7	18.80	6.35	16.19	18.45	20.83	0.275
	Other	15	20.50	5.19	16.50	20.98	22.62	
Left masseter	Rotary	7	16.07	3.83	13.13	17.34	19.73	0.148
	Other	15	20.07	5.90	14.77	19.07	24.23	
Right anterior temporalis muscle	Rotary	7	15.00	4.86	12.94	14.14	18.95	0.062
	Other	15	21.09	7.04	15.55	20.64	24.33	
Left anterior temporalis muscle	Rotary	7	18.48	4.95	14.01	20.37	22.55	0.072
	Other	15	23.53	5.70	18.86	23.57	25.58	
**Surface electromyography spontaneous chewing (% MVC in µV)**								
Right masseter	Rotary	7	8.35	2.83	6.14	7.48	9.89	0.698
	Other	15	9.07	3.45	6.42	8.01	12.35	
Left masseter	Rotary	7	7.82	4.08	5.64	6.40	9.13	0.192
	Other	15	9.43	3.27	6.66	9.32	12.31	
Right anterior temporalis muscle	Rotary	7	8.41	3.15	6.53	7.34	11.07	0.751
	Other	15	9.38	3.28	6.77	9.35	10.60	
Left anterior temporalis muscle	Rotary	7	11.00	3.09	7.15	11.15	14.29	0.503
	Other	15	10.17	3.12	7.05	10.99	13.03	
**Surface electromyography instructed chewing right (% MVC in µV)**								
Right masseter	Rotary	7	10.49	3.90	6.72	13.06	13.45	0.972
	Other	15	10.89	4.63	7.52	9.82	12.22	
Left masseter	Rotary	7	6.65	3.49	3.36	6.03	7.85	0.418
	Other	15	7.67	3.19	4.49	7.36	10.46	
Right anterior temporalis muscle	Rotary	7	8.26	2.79	5.42	8.26	11.00	0.275
	Other	15	9.48	2.71	7.13	9.15	11.34	
Left anterior temporalis muscle	Rotary	7	10.34	3.49	5.91	11.99	12.93	0.698
	Other	15	10.28	3.21	7.90	10.44	12.75	
**Surface electromyography instructed chewing left (% MVC in µV)**								
Right masseter	Rotary	7	8.02	3.60	4.91	9.70	11.21	0.805
	Other	15	7.68	3.73	5.12	6.50	10.23	
Left masseter	Rotary	7	9.86	4.71	4.58	9.36	14.97	0.378
	Other	15	11.47	3.89	9.53	9.85	15.18	
Right anterior temporalis muscle	Rotary	7	8.21	3.21	4.63	8.59	10.48	0.597
	Rotary	15	8.78	3.27	5.91	10.30	11.70	
Left anterior temporalis muscle	Rotary	7	12.04	4.86	7.63	13.53	15.19	0.566
	Other	15	10.94	3.08	8.63	11.34	13.34	

¥ Mann-Whitney U test;

A asymmetrical distribution of variables (Shapiro Wilk; p<0.05)

Caption: MVC = maximum voluntary contraction; µV = micro volts; SD = standard deviation

## Appendix B

**Table S2:** Measures of central tendency and variability of the masseter and anterior temporalis muscles at rest (10s), and during maximum intercuspation, spontaneous chewing and chewing on the right and left sides, with and without canine-guided occlusion

**Tasks**	**Descriptive measures**													
Guided occlusion	**RCGO**							**LCGO**						
	**n**	**X**	**DP**	**Quartile**			**p^¥^**	**n**	**X**	**DP**	**Quartile**			**p** ^¥^
				**1**	**2**	**3**					**1**	**2**	**3**	
**Surface electromyography at rest (% MVC in µV)**														
**Right masseter**														
With	12	0.92	0.79	0.32	0.64	1.23	0.060	12	0.90	0.78	0.32	0.68	1.05	0.070
Without	10	1.27	0.49	0.95	1.10	1.68		10	1.28	0.50	0.93	1.22	1.68	
**Left masseter**														
With	12	0.82	0.73	0.41	0.62	0.81	0.012	12	0.84	0.73	0.41	0.62	0.95	0.029
Without	10	1.45	0.83	0.80	1.15	2.16		10	1.43	0.84	0.80	1.07	2.16	
**Right anterior temporalis muscle**														
With	12	1.52	0.85	1.00	1.21	1.94	0.065	12	1.53	0.90	0.94	1.20	1.94	0.035
Without	10	2.14	0.87	1.42	2.23	2.72		10	2.13	0.81	1.42	2.09	2.72	
**Left anterior temporalis muscle**														
With	12	2.10	1.71	0.80	1.27	3.38	0.553	12	1.84	1.56	0.80	1.27	2.63	0.086
Without	10	2.46	1.26	1.82	2.41	2.89		10	2.78	3.31	2.13	2.60	3.61	
**Surface electromyography maximum intercuspation (% MVC in µV)**														
**Right masseter**														
With	12	20.88	6.04	16.25	19.39	22.38	0.947	12	19.81	4.39	16.25	19.39	22.06	0.742
WIthout	10	18.86	4.81	15.36	20.56	22.38		10	20.14	6.83	15.36	20.56	23.28	
**Left masseter**														
With	12	18.40	6.04	14.46	17.09	21.09	0.429	12	17.43	3.51	14.72	17.53	20.08	0.291
Without	10	19.28	5.23	14.63	19.35	24.31		10	20.45	7.21	14.39	21.87	24.64	
**Right anterior temporalis muscle**														
With	12	17.90	7.13	13.31	14.94	18.84	0.166	12	18.62	7.32	13.24	15.84	22.99	0.391
Without	10	20.65	6.76	17.12	21.18	23.59		10	19.78	6.79	15.00	20.72	22.68	
**Left anterior temporalis muscle**														
With	12	21.71	6.76	16.72	19.04	25.03	0.356	12	21.16	5.99	16.72	19.80	23.62	0.262
Without	10	22.17	4.96	20.48	22.87	25.16		10	22.83	5.91	20.10	22.87	26.39	
**Surface electromyography spontaneous chewing (% MVC in µV)**														
**Right masseter**														
With	12	8.44	3.14	6.18	7.67	11.06	0.429	12	8.44	3.22	6.18	7.02	11.44	0.291
Without	10	9.33	3.40	6.67	8.98	12.44		10	9.32	3.31	6.80	8.23	12.44	
**Left masseter**														
With	12	8.35	3.49	6.16	6.89	10.57	0.291	12	8.14	3.66	5.75	6.89	10.53	0.166
Without	10	9.59	3.65	6.45	9.92	12.88		10	9.84	3.32	7.03	10.01	12.89	
**Right anterior temporalis muscle**														
With	12	8.70	2.14	6.87	8.92	10.81	0.843	12	8.52	2.45	6.69	8.92	10.81	0.644
Without	10	9.52	4.22	6.35	8.79	13.12		10	9.74	3.95	6.72	8.79	13.12	
**Left anterior temporalis muscle**														
With	12	10.72	2.83	8.29	10.50	13.48	0.644	12	10.26	3.17	7.42	10.50	13.32	0.742
Without	10	10.10	3.43	6.88	11.50	13.10		10	10.66	3.07	7.08	11.89	13.13	
**Surface electromyography instructed chewing right (% MVC in µV)**														
**Right masseter**														
With	12	10.61	4.92	6.90	9.73	13.39	0.742	12	10.99	5.06	6.90	10.65	14.26	0.843
Without	10	10.95	3.73	7.43	10.30	13.43		10	10.49	3.49	7.70	9.78	12.43	
**Left masseter**														
With	12	7.14	3.58	4.37	6.37	9.22	0.598	12	7.20	3.60	4.24	6.91	9.22	0.692
Without	10	7.59	2.95	4.39	7.95	10.60		10	7.53	2.93	4.79	7.37	10.60	
**Right anterior temporalis muscle**														
With	12	9.22	2.87	7.21	8.62	11.26	0.947	12	9.30	2.75	7.33	8.62	11.26	0.792
Without	10	8.93	2.71	6.63	9.04	11.44		10	8.84	2.85	6.59	9.04	11.44	
**Left anterior temporalis muscle**														
With	12	10.72	3.22	8.15	11.61	13.64	0.429	12	10.41	3.49	6.45	11.61	13.64	0.742
Without	10	9.79	3.31	6.91	9.39	12.11		10	10.17	3.04	7.74	9.96	12.11	
**Surface electromyography instructed chewing left (% MVC in µV)**														
**Right masseter**														
With	12	6.98	3.18	5.02	5.83	10.97	0.429	12	6.73	3.30	4.00	5.80	10.83	0.187
Without	10	8.76	4.01	5.28	9.09	11.00		10	9.06	3.71	5.56	9.09	11.00	
**Left masseter**														
With	12	10.30	3.52	8.71	9.71	11.71	0.429	12	10.39	4.46	7.06	9.82	14.22	0.742
Without	10	11.75	4.83	8.07	14.11	15.40		10	11.64	3.80	9.35	11.67	15.21	
**Right anterior temporalis muscle**														
With	12	8.23	2.40	6.04	8.32	10.44	0.468	12	7.87	2.89	5.06	8.32	10.44	0.187
Without	10	9.05	4.02	4.51	10.71	12.61		10	9.47	3.46	6.07	10.71	12.61	
**Left anterior temporalis muscle**														
With	12	11.62	2.55	8.81	11.91	13.59	0.644	12	11.48	4.05	8.06	11.91	13.97	0.792
Without	10	10.89	4.79	6.68	10.40	15.29		10	11.06	3.32	8.21	10.79	14.01	

¥ Mann-Whitney U test comparing lateral excursions with and without canine-guided disclusion

Caption: MVC = maximum voluntary contraction; µV = micro volts; A = asymmetrical distribution of variables (Shapiro Wilk; p<0.05); RCGO = right canine-guided occlusion; LCGO = left canine-guided occlusion; X = mean, SD = standard deviation, Min = minimum; Max = maximum

## Appendix C

**Table S3:** Measures of central tendency and variability of the masseter and anterior temporalis muscles at rest (10s), and during maximum intercuspation, spontaneous chewing and chewing on the right and left sides, with and without occlusal interference on the working side

**Tasks**	**Descriptive measures**													
Guided occlusion	OIRS							OILS						
	**n**	**X**	**DP**	**Quartile**			**p¥**	**n**	**X**	**DP**	**Quartile**			**p** ^¥^
				**1**	**2**	**3**					**1**	**2**	**3**	
**Surface electromyography at rest (% MVC in µV)**														
**Right masseter**														
With	9	1.32	0.78	0.74	1.09	2.00	0.204	8	1.49	0.72	1.01	1.38	2.17	0.026
Without	13	0.91	0.58	0.44	0.82	1.34		14	0.84	0.55	0.37	0.78	1.16	
**Left masseter**														
With	9	1.28	0.96	0.65	0.81	2.05	0.367	8	1.55	0.92	0.79	1.30	2.57	0.034
Without	13	0.99	0.73	0.48	0.75	1.35		14	0.85	0.67	0.46	0.75	0.90	
**Right anterior temporalis muscle**														
With	9	2.29	0.98	1.47	2.10	3.32	0.021	8	2.35	1.05	1.47	2.05	3.48	0.029
Without	13	1.47	0.69	0.96	1.08	2.23		14	1.49	0.66	0.97	1.21	2.12	
**Left anterior temporalis muscle**														
With	9	2.90	1.80	1.44	2.65	4.35	0.171	8	2.77	1.91	1.39	2.49	4.53	0.453
Without	13	1.83	1.12	0.85	1.98	2.66		14	1.98	1.18	0.89	2.02	3.05	
**Surface electromyography maximum intercuspation (% MVC in µV)**														
**Right masseter**														
With	9	20.82	4.53	17.81	20.83	22.25	0.526	8	19.62	6.26	15.00	20.56	22.60	0.946
Without	13	13.37	6.17	15.75	19.37	22.41		14	20.16	5.24	16.35	19.39	22.30	
**Left masseter**														
With	9	19.23	4.05	14.61	19.73	22.77	0.483	8	18.38	5.19	14.34	19.40	23.27	0.891
Without	13	18.50	6.57	13.80	17.34	23.66		14	19.04	5.96	14.46	17.53	23.48	
**Right anterior temporalis muscle**														
With	9	19.43	6.53	13.75	18.50	22.07	0.867	8	18.89	7.83	12.59	19.53	22.71	0.838
Without	13	18.96	7.47	13.26	17.34	23.40		14	19.30	6.69	13.34	17.92	22.93	
**Left anterior temporalis muscle**														
With	9	20.22	3.33	18.14	20.51	22.87	0.271	8	19.51	4.96	15.31	20.84	22.22	0.275
Without	13	23.10	7.03	17.45	23.38	27.48		14	23.30	6.07	18.17	22.97	26.53	
**Surface electromyography spontaneous chewing (% MVC in µV)**														
**Right masseter**														
With	9	10.32	3.49	7.94	11.65	13.37	0.057	8	10.48	3.74	7.12	11.80	13.70	0.076
Without	13	7.82	2.68	6.23	6.75	9.15		14	7.91	2.56	6.27	7.12	8.77	
**Left masseter**														
With	9	10.70	3.10	7.81	10.70	13.12	0.030	8	10.49	3.91	7.49	10.85	13.36	0.116
Without	13	7.68	3.38	4.95	6.58	10.53		14	8.02	3.09	5.97	6.89	9.97	
**Right anterior temporalis muscle**														
With	9	10.21	3.16	7.05	10.37	11.92	0.133	8	10.12	3.26	7.33	9.92	12.06	0.219
Without	13	8.29	3.09	6.12	8.02	10.42		14	8.47	3.11	6.32	8.26	10.67	
**Left anterior temporalis muscle**														
With	9	11.18	3.12	7.65	12.39	13.66	0.333	8	10.81	3.41	7.06	12.15	13.97	0.633
Without	13	9.92	3.03	7.12	10.01	12.64		14	10.23	2.95	7.97	10.50	13.13	
**Surface electromyography instructed chewing right (% MVC in µV)**														
**Right masseter**														
With	9	12.22	3.52	9.78	12.22	14.44	0.102	8	12.00	4.91	7.84	12.64	15.05	0.246
Without	13	9.75	4.66	6.94	7.88	12.06		14	10.06	4.43	7.25	9.63	11.97	
**Left masseter**														
With	9	8.53	3.69	5.09	8.46	12.22	0.217	8	8.71	3.99	4.58	8.96	12.79	0.219
Without	13	6.53	2.74	3.93	6.46	8.76		14	6.57	2.57	4.41	6.37	8.31	
**Right anterior temporalis muscle**														
With	9	10.82	2.49	8.68	11.08	12.54	0.021	8	10.16	2.58	7.85	11.04	12.21	0.133
Without	13	7.89	2.27	6.01	8.26	9.04		14	8.48	2.72	6.89	8.36	9.17	
**Left anterior temporalis muscle**														
With	9	10.51	2.16	8.91	10.44	12.46	0.867	8	10.12	2.80	7.66	9.96	12.70	0.946
Without	13	10.15	3.86	5.77	11.23	13.35		14	10.40	3.53	7.33	10.94	13.05	
**Surface electromyography instructed chewing left (% MVC in µV)**														
**Right masseter**														
With	9	8.09	2.93	5.30	8.54	11.02	0.526	8	8.55	2.71	5.44	9.09	11.12	0.375
Without	13	7.58	4.11	5.02	6.05	10.72		14	7.35	4.06	4.76	5.83	10.48	
**Left masseter**														
With	9	12.14	2.68	9.54	11.96	15.08	0.367	8	11.78	3.90	9.40	12.87	15.13	0.585
Without	13	10.14	4.82	5.58	9.79	15.10		14	10.49	4.32	8.03	9.71	14.77	
**Right anterior temporalis muscle**														
With	9	9.30	2.79	6.51	10.30	11.37	0.483	8	8.95	3.25	5.23	9.81	11.54	0.733
Without	13	8.12	3.46	4.80	8.05	11.39		14	8.40	3.25	5.67	8.32	11.08	
**Left anterior temporalis muscle**														
With	9	11.53	2.75	9.06	12.35	13.67	0.815	8	10.99	3.26	7.69	11.60	13.45	0.682
Without	13	11.12	4.27	7.83	11.34	14.65		14	11.46	3.97	8.14	11.40	14.38	

¥ Mann-Whitney U test comparing lateral excursions with and without canine-guided disclusion

Caption: A = asymmetrical distribution of variables (Shapiro Wilk; p<0.05); OIRS = occlusal interference on the right working side; OILS = occlusal interference on the left working side; X = mean, SD = standard deviation; Min = minimum; Max = maximum

## Supplementary Material

Supplementary material accompanies this paper.

Supplementary chart 1: Study variables, variable definitions and unit of measurement

Supplementary chart 2: Study protocol flowchart

This material is available as part of the online article from https://doi.org/10.1590/2317-1782/e20240052en
