# Supplementary material for: Mastication and electrical activation in the masseter and anterior temporalis muscles of children and adolescents with osteogenesis imperfecta
Source: Codas. 2025 Jan 27;37(1):e20240052. doi: 10.1590/2317-1782/e20240052en (PMC11781364; doi:10.1590/2317-1782/e20240052en)
Supplement: Supplementary chart 1: [file codas-37-1-e20240052-suppl01.pdf]

Supplementary chart 1: Study variables, variable definitions and unit of measurement

Variables	Definition	Unit of measurement
At rest	Task rest[19]	µV
	The electromyographic signals normalized	
Maximum intercuspation	Task maximum intercuspation[19]	µV
	The electromyographic signals normalized	
Spontaneous chewing	Task spontaneous chewing[19]	µV
	The electromyographic signals normalized	
Instructed chewing right	Task instructed chewing right[19]	µV
	The electromyographic signals normalized	
Instructed chewing left	Task instructed chewing left[19]	µV
	The electromyographic signals normalized	
Right masseter	Muscle right masseter[19]	µV
	The electromyographic signals normalized	
Left masseter	Muscle left masseter[19]	µV
	The electromyographic signals normalized	
Right anterior temporalis muscle	Muscle right anterior temporalis muscle[19]	µV
	The electromyographic signals normalized	
Left anterior temporalis muscle	Muscle left anterior temporalis muscle[19]	µV
	The electromyographic signals normalized	
Muscle activation index	Corresponds to the percentage index of muscle activation[21]	%
Muscle asymmetry index	Indicate a predominance of muscle activation[22]	%
Total asymmetry index	Total percentage index of muscular asymmetry	%
Masseter muscle asymmetry index	Masseter percentage index of muscular asymmetry	%

Anterior temporalis muscle asymmetry	Anterior temporalis percentage index of muscular asymmetry	%

Supplementary chart 2: Study protocol flowchart

**Step One**
After a routine consultation at the outpatient clinic, individuals were invited to participate in the study.
**Step Two**
Those who accepted were referred to perform the study evaluations.
**Step Three**
Clinical evaluation was performed the items guided occlusion and occlusal interferences during lateral excursions were analyzed.
**Step Four**
An orofacial myofunctional evaluation of chewing simultaneously with surface electromyography during chewing tasks. The chewing was performed with video camera recording for later analysis.
